# Smoking and Breast Cancer Recurrence after Breast Conservation Therapy

**DOI:** 10.1155/2014/327081

**Published:** 2014-02-17

**Authors:** Jennifer D. Bishop, Brigid K. Killelea, Anees B. Chagpar, Nina R. Horowitz, Donald R. Lannin

**Affiliations:** Department of Surgery and Yale Comprehensive Cancer Center, Yale University School of Medicine, New Haven, CT 06520, USA

## Abstract

*Background*. Prior studies have shown earlier recurrence and decreased survival in patients with head and neck cancer who smoked while undergoing radiation therapy. The purpose of the current study was to determine whether smoking status at the time of partial mastectomy and radiation therapy for breast cancer affected recurrence or survival. *Method*. A single institution retrospective chart review was performed to correlate smoking status with patient demographics, tumor characteristics, and outcomes for patients undergoing partial mastectomy and radiation therapy. *Results*. There were 624 patients who underwent breast conservation surgery between 2002 and 2010 for whom smoking history and follow-up data were available. Smoking status was associated with race, patient age, and tumor stage, but not with grade, histology, or receptor status. African American women were more likely to be current smokers (22% versus 7%, *P* < 0.001). With a mean follow-up of 45 months, recurrence was significantly higher in current smokers compared to former or never smokers (*P* = 0.039). In a multivariate model adjusted for race and tumor stage, recurrence among current smokers was 6.7 times that of never smokers (CI 2.0–22.4). *Conclusions*. Although the numbers are small, this study suggests that smoking may negatively influence recurrence rates after partial mastectomy and radiation therapy. A larger study is needed to confirm these observations.

## 1. Introduction

Radiation therapy is a key component of breast conservation therapy for *in situ* or invasive breast cancers. Fisher's landmark NSABP B-06 study [1] showed at twenty-year follow-up a significant decrease in ipsilateral breast tumor recurrence from 39.2% to 14.3% (*P* < 0.001) when lumpectomy patients were treated postoperatively with whole breast irradiation. The benefit of radiation was seen regardless of nodal status. The Early Breast Cancer Trialists' Collaborative Group's meta-analysis [2] of 7300 women treated with breast-conserving surgery and postoperative radiotherapy also showed a decrease in ipsilateral breast tumor recurrence over 5 years from 26% without radiotherapy to 7% with radiotherapy (*P* = 0.0002). In contrast to the NSABP trial, this study also showed a 15-year breast cancer mortality risk reduction from 35.9% without radiation to 30.5% with radiation (*P* = 0.005). Therefore, the necessity of radiation in association with breast conservation surgery is widely accepted.

Radiation causes DNA damage through the formation of free radicals which cause irreparable damage to cancer cells. A major limitation, however, in radiation therapy for solid tumors is hypoxia. Oxygen is a potent radiosensitizer and a key component of radiation damage. Cigarette smoking is known to cause tissue hypoxia. In 1993, Browman and colleagues [3] showed that head and neck cancer patients who continued to smoke during radiation therapy had lower rates of response and shorter disease free survival and overall survival than patients who did not smoke during radiation. However, this phenomenon has never been studied in patients undergoing radiation therapy for breast cancer. The purpose of this study was to compare the recurrence rates for patients who smoked or did not smoke during radiation therapy after breast conservation surgery.

## 2. Methods

After Institutional Review Board approval, the prospectively collected database at The Breast Center Smilow Cancer Hospital at Yale New Haven was queried to capture cases of *in situ* and invasive breast cancers diagnosed between the years 2002 and 2010 treated with partial mastectomy followed by radiation therapy. Patient demographics, tumor characteristics, further treatment including adjuvant systemic and radiation therapy, recurrence, and survival were obtained from both the database and the hospital tumor registry. Patient charts were then reviewed to ascertain smoking status from the patient's intake history form or from physician-documented histories or initial consultations. Patient smoking history was categorized as never, former, current, or not available. Former smokers were defined by the provider or patient through subjective history and the duration since last tobacco use was unknown.

Patients were treated in standard fashion with partial mastectomy and postoperative whole breast, external beam radiation therapy. Treatment of the axillary field was at the discretion of the radiation oncologist and varied on a case-by-case basis depending on the burden of lymph node disease. The majority of patients were treated with standard 2-tangent whole breast irradiation if less than 4 lymph nodes were involved or 4 field, if more than 4 lymph nodes were involved. Patients who did not undergo radiation or underwent mastectomy were excluded from this study.

Of 1166 charts reviewed on patients undergoing partial mastectomy, 624 had information regarding smoking status and follow-up documented and were included in this study. Five of the included patients had follow-up at other institutions and were known to have had recurrence but it was not known whether the recurrence was local or distant. Pathologic assessment of surgical cases was performed by dedicated breast pathologists. All cases of ductal intraepithelial neoplasia (DIN) were included, except for grade 1 DIN less than 2 mm in size, which is considered atypical ductal hyperplasia (ADH). Lobular carcinoma *in situ* was not included in this analysis.

Statistical analysis was performed with SPSS software version 19. Categorical data were compared using chi-square tests and Kaplan-Meier curves were constructed and compared with log rank (Mantel-Cox) tests for recurrence-free survival. Multivariable models of factors affecting recurrence were analysed with Cox regression.

## 3. Results

Of the 624 patients in the study, 52 (8.3%) were current smokers at the time of diagnosis, 196 (31.4%) were former smokers, and 376 (60.3%) were never smokers. Patient demographic and tumor characteristics by smoking history are shown in Table 1. Smoking status was associated with race, patient age, and tumor stage, but not with grade, histology, or receptor status. African American women were significantly more likely to be current smokers than other racial/ethnic groups, and current smokers were significantly younger than nonsmokers. In addition, there were significant differences by tumor stage. Current smokers were more likely to have stage 0 disease (DCIS) or stage 2 disease, whereas never smokers were most likely to have stage 1 disease. There was a trend for current smokers to have less infiltrating lobular cancer, but this was not statistically significant. There was no difference in estrogen receptor, progesterone receptor, or Her2/neu receptor expression (or any molecular subtype combination such as ER/PR −, Her2 +, or triple negative) by smoking history.

**Figure 1:**
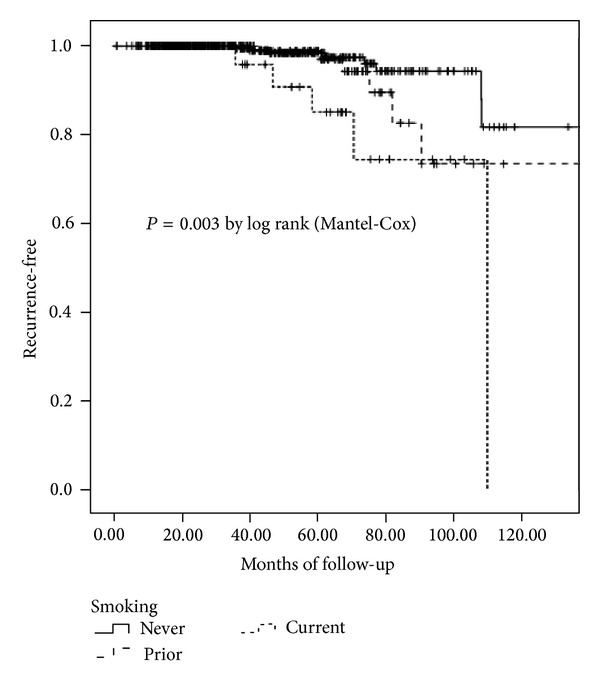
Kaplan-Meier plot of recurrence-free survival by smoking status.

At the time of analysis, the mean follow-up was 45 months and the median follow-up was 40 months (range 5–130). There were 22 total recurrences; the type of recurrence by smoking history is shown in Table 2. There were too few cases to analyze the type of recurrence, but any recurrence was significantly more frequent in current smokers than in never smokers (*P* = 0.039).  Figure 1 shows Kaplan-Meier curves for recurrence-free survival. Current smokers tended to recur sooner than never smokers, and prior smokers were intermediate between the two (*P* = 0.003 by Log rank). Multivariable Cox regression models were constructed using race, age, stage, and smoking status and, as shown in Table 3, both stage and smoking status remained significantly associated with recurrence. When adjusted for race, age, and tumor stage, current smokers were 6.7 times more likely to develop a recurrence than never smokers (95% CI: 2.0–22.4). There was no statistically significant difference, however, in overall survival by smoking status.

## 4. Discussion

This is the first study to show that, among breast cancer patients treated with partial mastectomy and radiation therapy, current smokers have a significantly higher recurrence rate than prior smokers or never smokers. Although the numbers are small, almost 10% of current smokers had a documented recurrence at a mean follow-up of 45 months, compared with less than 4% for never and former smokers (2.7 and 3.6%, resp.). There was a tendency for current smokers to have more stage 2 disease whereas never smokers had more stage 1 disease. In a multivariable model, however, smoking influenced recurrence independent of stage.

The key mechanism of action of radiation involves direct DNA damage through formation of free radicals which cause irreparable damage, slowing the growth of cancers or causing direct cell death. As cancer cells are usually dividing at a faster rate than normal cells, they are more directly affected by radiation damage. Oxygen is a potent radiosensitizer and a key component of radiation damage. When cancer cells are in a hypoxic milieu, they are thought to be less susceptible to radiation-induced damage. The results of the current study would seem to support the hypothesis of Browman and colleagues [3] who showed similar results for head and neck cancer patients who continued to smoke through radiation therapy.

A review by Holmes and colleagues of the Nurses' Health Study in 2007 found on multivariate analysis that current smokers had a 43% increase in adjusted relative risk of death from any cause [4]. Current and past smokers had higher rates of death from lung cancer, COPD, and other lung diseases, but no direct increase in mortality from breast cancer. However, Holmes' study evaluated all methods of treatment for breast cancer in this diverse patient population of 5,056 patients. Less than half of this group received breast conservation therapy with lumpectomy and radiation. There was no significant difference in mortality among smokers compared with nonsmokers either with or without radiation.

With regard to breast cancer and smoking status, prior studies have shown a decreased overall survival of smokers. Calle and colleagues' 1994 epidemiological study [5] followed 676,530 female volunteers prospectively from 1982 to 1988 as part of the Cancer Prevention Study II through the American Cancer Society. During the study period, they found 880 deaths from breast cancer with an increased relative risk of death for current smokers (RR = 1.26; 95% CI: 1.05–1.50). Furthermore, breast cancer mortality increased with total number of years smoked and number of cigarettes per day (*P* = 0.0096 and *P* = 0.0003, resp.). Types of treatment, including surgery, drug therapy, and radiation therapy, were not included in this study. Manjer et al. [6] followed 792 women diagnosed with breast cancer in Sweden from 1977 to 1986. Relative risk of mortality from breast cancer compared with never smokers was 1.44 for current smokers (95% CI: 1.01 to 2.06) and 1.13 (95% CI: 0.66 to 1.94) for prior smokers. All-cause mortality adjusted for age was 1.46 for smokers (95% CI: 1.15 to 1.86) compared to never smokers. This increased mortality was significant even when adjusted for age and stage at diagnosis. Data from 12,989 female patients in Memorial Sloan-Kettering Cancer Center's tumor registry was analyzed by Yu and colleagues [7] in 1997, and a history of smoking carried a risk ratio of 1.43 for all-cause mortality. This effect was seen not only for breast cancer, but also for oral and pancreatic cancers.

It is interesting to speculate whether the results of the current study may have implications for breast cancer on a population level. A major epidemiologic problem has been to explain the increased mortality from breast cancer among African American women [8]. Most studies have focused either on reason for later stage of diagnosis [9] or on biological factors leading to aggressive tumor behavior [10, 11]. However, the finding that African American women are more likely to be smokers, and that this may be detrimental, opens up a new avenue for investigation. Prior epidemiologic studies have shown interesting molecular changes associated with African American race, smoking status, and breast cancer development [12]. Current smokers have also been shown to have increased rates of p53 mutations, independent of stage at diagnosis [13], which has been shown on multivariate analysis to be a predictor of poor prognosis [14].

Limitations of this study include the small number of current smokers and the uncertain location of recurrence in five patients as documented by the tumor registry. Follow-up or smoking status was not available for 542 patients of the initial 1166 lumpectomy patients identified in our database, because the patient intake form was incomplete or because some patients only had consultations or part of their care provided at our center. In addition, it is unknown whether some patients may have stopped smoking after the diagnosis was made. Future directions would include looking at a larger study population, examining postmastectomy radiation patients to see if they had similar outcomes and examining other concomitant epigenetic and environmental factors that may be confounding the data, such as BMI and socioeconomic status.

This study leaves many questions unanswered. If the results are really due to tissue hypoxia during radiation, one would expect this to primarily affect local and not distant recurrence. Similarly mastectomy patients who do not receive radiation should not be affected. It is unclear at this time whether the results of this study should influence our treatment of breast cancer. Although other factors could be confounding the findings, the results would suggest that smokers should be actively counseled to quit smoking at least through the duration of therapy. This could potentially even raise the question of not offering breast conservation therapy to active smokers, as they may be less likely to benefit from the effects of radiation therapy. Clearly, larger more definitive studies should be performed.

## Figures and Tables

**Table 1:** Demographic and tumor characteristics of sample by smoking history.

	Smoking history—number (%)			*P* value
	Never (*n* = 376)	Prior (*n* = 196)	Current (*n* = 52)	
Race				<0.001
White	288 (77%)	160 (81%)	31 (60%)	
Black	43 (11%)	21 (11%)	17 (33%)	
Asian	13 (3%)	1 (1%)	0 (0%)	
Hispanic	21 (6%)	5 (3%)	4 (7%)	
Other	11 (3%)	9 (5%)	0 (0%)	
Age (mean ± SD)	60 ± 13	62 ± 11	57 ± 11	0.007
Stage				0.004
0	96 (26%)	55 (28%)	20 (38%)	
1	211 (57%)	92 (47%)	16 (31%)	
2	57 (15%)	43 (22%)	16 (31%)	
3	8 (2%)	6 (3%)	0 (0%)	
Histology				0.08
Infiltrating ductal	195 (52%)	110 (56%)	27 (52%)	
DCIS	95 (25%)	53 (27%)	21 (40%)	
Infiltrating lobular	21 (6%)	13 (7%)	1 (2%)	
Mixed ductal/lobular	15 (4%)	6 (3%)	0 (0%)	
Other	50 (13%)	14 (7%)	3 (6%)	
ER^a^ (% positive)	86% (287/334)	84% (144/171)	86% (37/43)	0.87
PR^a^ (% positive)	78% (253/323)	76% (129/170)	83% (35/42)	0.56
Her2/neu^a^ (% positive)	16% (34/206)	10% (12/120)	17% (5/29)	0.24

^a^ER/PR only obtained on DCIS after 2005 and Her2/neu obtained on invasive cases only after 2005.

**Table 2:** Recurrence by smoking status.

	Type recurrence			Any recurrence	No recurrence	Total
	Local	Distant	Unknown			
Never smoker	4	5	1	10 (2.7%)	366 (97.3%)	376 (100%)
Prior smoker	3	1	3	7 (3.6%)	189 (96.4%)	196 (100%)
Current smoker	3	1	1	5 (9.6%)	47 (90.4%)	52 (100%)

Total	10	7	5	22 (3.5%)	602 (96.5%)	624 (100%)

*P* = 0.039 for any recurrence versus no recurrence.

**Table 3:** Multivariable Cox regression for recurrence-free survival.

	Hazard ratio (95% confidence interval)	*P* value
Smoking		
Non smoker	Reference	
Prior smoker	1.43 (0.48–4.30)	0.524
Current smoker	6.69 (2.00–22.42)	0.002
Stage		
0	Reference	
1	1.28 (0.28–5.75)	0.747
2	10.66 (2.46–46.18)	0.002
3	20.89 (1.75–249.18)	0.016
